# Physical Medicine & Rehabilitation Physicians’ Collective Insight Points to Psychosocial Factors as Central to Understanding Low Back Pain.

**DOI:** 10.51894/001c.143506

**Published:** 2025-09-30

**Authors:** Tray P. Heliin, Angela S Lee, Kai Francisco, John M Popovich

**Affiliations:** 1 College of Osteopathic Medicine Michigan State University

## 03

### INTRODUCTION

Chronic low back pain (CLBP) is a multi-faceted condition which draws many contributions from both biological and psychological factors. The etiology is not fully understood, and the management spans many medical specialties. Physical Medicine & Rehabilitation (PM&R) physicians frequently treat patients with CLBP, but little research has been done regarding their perspectives on contributing factors to the condition.

### OBJECTIVES

The objective of this study is to identify the most important perceived factors contributing to CLBP by PM&R trained physicians. We hypothesize that PM&R physicians will attribute more importance in a biological/structural approach rather than a psychological/social.

### METHODS

PM&R physicians with experience treating patients with CLBP were recruited for this study. Participants underwent semi-structured 1-on-1 interviews to create individual fuzzy cognitive maps (FCMs) using online software (mentalmodeler.com). Each FCM represents a participant’s “mental model” regarding CLBP. After all FCMs were completed, they were aggregated to form a representative meta-model of all participants. Ten categories were incorporated in the meta-model (Biomechanical, Comorbidities, Individual, Behavioral/Lifestyle, Nociception Detection and Processing, Psychological, Social/Work/Contextual, Tissue Injury or Pathology, Outcomes, and Treatment/Intervention) which all have nodes that contribute to them. The sum of centrality (Sc) is calculated for each category, with a larger Sc indicating greater importance attributed to the category.

### RESULTS

Twenty-six PM&R physicians, including 14 with fellowship-training in Sports Medicine, Pain Medicine, or Interventional Spine, participated. FCMs from all participants consisted of 634 nodes and 1398 connections. The aggregated meta-model consisted of 112 unique nodes and 572 connections, underscoring its complexity and comprehensive structure. The category that had the highest Sc was Psychological (23%) followed by Social/Work/Contextual (22%), and Behavioral/Lifestyle (17%). The top interventions listed in the Treatment/Intervention category included Physical Therapy, Exercise Therapy, Spinal Injections, and Psychological Interventions.

### CONCLUSION

Amongst PM&R physicians, psychosocial factors are viewed as central contributors to understanding CLBP. While the most common treatments targeted biomechanical and tissue categories, which may not specifically address psychosocial factors, psychosocial factors emerged as central to physicians’ understanding of CLBP. These findings highlight PM&R physicians’ distinct role and emphasize a holistic approach that considers multiple factors in developing individualized care for patients with CLBP.

